# Hybrid Cluster-Continuum Method for Single-Ion Solvation Free Energy in Acetonitrile Solvent

**DOI:** 10.1021/acs.jpca.4c03593

**Published:** 2024-07-25

**Authors:** Josefredo R. Pliego

**Affiliations:** Departamento de Ciências Naturais, Universidade Federal de São João del-Rei, São João del-Rei, Minas Gerais 36301-160, Brazil

## Abstract

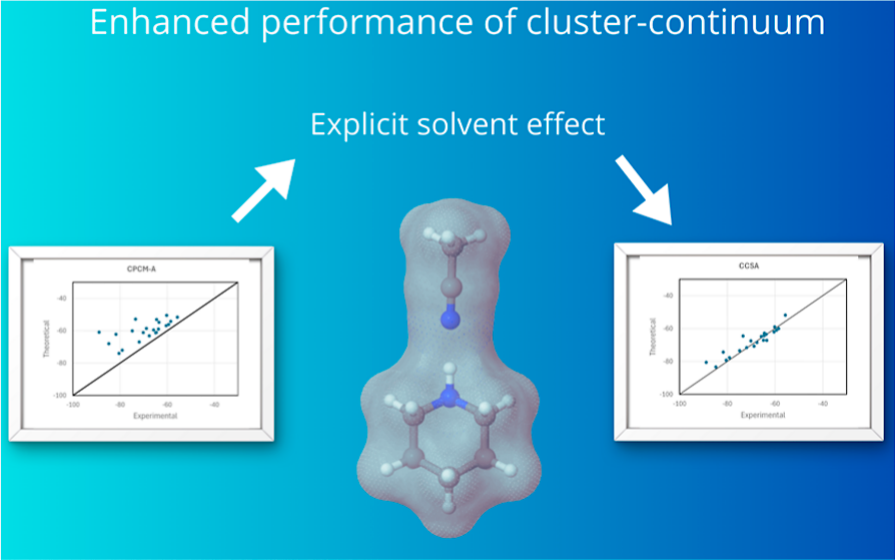

A new hybrid discrete-continuum approach named the cluster-continuum static approximation (CCSA) has been proposed for acetonitrile solvent. The continuum part uses the conductor-like polarizable continuum model for electrostatic and a surface area-dependent term for nonelectrostatic solvation. The CCSA includes only one explicit acetonitrile solvent molecule and a damping function, which makes the CCSA method reduce to pure continuum solvation in the case of weaker potential of mean force for solute–solvent interaction. The performance of the model was tested for 22 anions and 22 cations, including challenge species that cannot be adequately described by pure continuum solvation. A comparison was done with the widely used solvent model density (SMD) model. For anions, the CCSA reduces to pure continuum solvation and the method has the same performance as the SMD model, with a standard deviation of the mean signed error (SD-MSE) of 2.7 kcal mol^–1^ for both models. However, the CCSA method for cations considerably outperforms the SMD model, with an SD-MSE of 3.3 kcal mol^–1^ for the former and 8.4 kcal mol^–1^ for the latter. The method can be automated, and the present study suggests that continuum solvation models could be parameterized taking into account the explicit solvation as proposed in this work.

## 1 Introduction

Modeling the solvation phenomenon remains a very active research topic in theoretical chemistry.^1^ Despite the variety of models and their evolution over the past decades, accurate modeling of ionic reactions in the solution phase remains a hard task, usually requiring expensive QM/MM methods for more reliable free energy barriers.^2−4^ Widely used and practical continuum solvation models have limited accuracy.^5−7^ Thus, while gas-phase free energies of chemical reactions can be determined with chemical accuracy, resulting in reliable rate constants for small molecules,^8,9^ the solution phase processes present substantially higher uncertainty. For example, widely used solvation models such as solvent model density (SMD) can lead to substantial error in some cases, as the process of formation of a pair of ions in the solution phase.^5,10^ Considering that the explicit solvent approach as QM/MM or full quantum-mechanical methods are very expensive, improvements in continuum solvation models seem the most viable alternative. An approach is the inclusion of explicit solvent molecules into the continuum, leading to cluster-continuum methods.^11,12^

In the continuum solvation approach,^13−17^ contributions to the solvation free energy as cavity formation and dispersion energy can be reasonably well described by adequately parameterized models.^18−21^ Adding these terms to the electrostatic contribution usually leads to efficient and relatively accurate solvation models. For example, the continuum solvation models SMD, SM12, and COSMOS-RS have a relatively good performance for the solvation of neutral species.^21−25^ For the set of 274 neutral solutes in an aqueous solution, the mean unsigned error (MUE) of SMD is in the range of 0.59 to 0.94 kcal mol^–1^ depending on the electronic density used.^21^ For a set of 2072 neutral solutes in a nonaqueous solvent, the error is in the range of 0.64 to 0.79 kcal mol^–1^. In the same way, the COSMO-RS model performs very well, with a MUE of 0.48 kcal mol^–1^ for all sets of 2346 solutes.^23^ Nevertheless, in more challenging situations such as more complex molecules, the error is higher. In the SAMPL4 data set of 47 complex multifunctional compounds, the root mean squared error (RMSE) in COSMO-RS was 1.46 kcal mol^–1^. For 56 molecules of a subset of the SAMPL1 data set, the RMSE for SMD was reported as 2.5 kcal mol^–1^.^26^

In the interesting case of single ion solvation, the performance of continuum models worsens considerably. For example, an analysis of the hydration free energy of 112 ions led to RMSE in the range of 5.6 to 8.0 kcal mol^–1^ for the SMD model, depending on the used electronic structure method, with the best coefficient of determination (R^2^) calculated value to be 0.86.^27^ An approach to include strong solute–solvent interaction in the continuum model is the idea of field-extremum correction, where the highest electric field on the cavity surface of a molecule is used to introduce a correction to the free energy.^28−30^ Such an idea combined with continuum solvation contribution and terms for nonelectrostatic solvation led to the composite method for implicit representation of the solvent (CMIRS) by Pomogaeva and Chipman.^31−33^ The approach was parameterized for water, DMSO, acetonitrile, benzene, and cyclohexane^31−33^ and was further parameterized for methanol.^5^ The method was reparameterized by You and Herbert and tested for solvation free energy of neutrals and ions, leading to a MUE of 2.4 kcal mol^–1^ for ions in an aqueous solution.^34^ Similar approaches including field extremum correction as the xESE and uESE methods also work finer than pure continuum models for ions.^35,36^ More recently, a continuum solvation model (CSM) with variable atomic radii has been proposed.^37^ The performance of the COSMO-RS has also been tested for ions in aqueous, methanol, DMSO, and acetonitrile solvents, with an average absolute deviation going from 1.3 to 5.0 kcal mol^–1^, depending on the solvent, if a set of cations or anions are considered, and the electronic structure method used.^38^

Another widely used pathway for improving pure continuum models is by a hybrid discrete-continuum approach.^11^ The main advantage of explicit inclusion of solvent molecules is taking into account strong solute–solvent interaction, which is difficult to describe by pure continuum models and even a field-extremum approach. For example, the CMIRS model was not able to describe accurately ionic reactions in methanol solution, with a standard deviation of the mean signed error (SD-MSE) of the solvation free energies of 4.1 and 3.2 kcal mol^–1^ for anions and cations, respectively.^5^ On the other hand, the discrete-continuum approach could theoretically connect pure continuum solvation to pure discrete solvation. Thus, any level of accuracy could be obtained. Nevertheless, the process is not simple because adequate sampling of configurations and questions on how to restrict the solvent molecules close to the solute by theoretically sound method emerges.^39,40^ In many situations, the inclusion of explicit solvent molecules has been done without basic statistical mechanics support or adequate protocol. A theoretically sound method is the cluster-continuum quasichemical theory (CCQC), where the solute and some explicit solvent molecules are considered a chemical species.^12,41−43^ This approach works accurately when the solvent molecules are strongly bound to the solute^44^ and have been used to establish a bulk single-ion solvation free energy scale in solution in agreement with the TATB assumption for water, methanol, and DMSO solvents.^45,46^ The method also works for chemical reactions.^47,48^ Although useful, in the case of the solvent molecule more weakly bound to the central ion, the accuracy of the method decreases and a more general procedure is desirable.

More recently, Pliego has developed a cluster-expansion theory of the solvation free energy difference for explicit solvent and derived an approximated expression for a hybrid cluster-continuum method.^49^ Further simplification led to the cluster-continuum static approximation (CCSA),^49^ which was recently tested for single ion solvation in an aqueous solution.^10^ The method was used in conjunction with the SMD model and has presented a notable improvement in the solvation free energy in relation to the pure SMD model. Essentially, the method introduces a strong solute–solvent interaction by computing the potential of mean force for the addition of the solvent molecule to the solute and an additional integral term that was set as a constant. In this work, the objective is to further develop this approach and to apply it to the challenging solvation of ions in acetonitrile. We have used the bulk free energy scale in acetonitrile solvent as well as some solvation data reported by Carvalho and Pliego,^45^ the data available in the DISSOLVE database,^50^ and also new solvation data was generated for 8 multifunctionalized anions. A total of 22 cations and 22 anions with diverse functional groups were tested. For evaluating the improved performance of the CCSA method in relation to a nowadays widely used standard method, the SMD model was used as a reference. The comparison with the performance of the CCQC method was also done.

## 2 Methods

### 2.1 Theory

The hybrid approach proposed in this work uses a CSM and explicit inclusion of solvent molecules. In the continuum part, the conductor-like polarizable continuum model (CPCM) method^51^ with the solvent-excluded surface (SES) and a solvent radius (R_solv_) of 2.49 Å was used for calculating the electrostatic contribution to the free energy. This value is based on the density of acetonitrile and considers that the molecules occupy 75% of the volume, leading to a molecular volume of 65.0 Å^3^. The nonelectrostatic solvation contribution was described by a simple surface area-dependent term. The method was called CPCM-A, and the solvation free energy is given by the equation
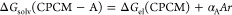
1where the area of the molecule (*Ar*) was calculated using the solvent-excluded surface (SES) and the α_A_ parameter (atomic surface tension) was fitted to reproduce the Δ*G*_solv_ of neutral species. Some continuum solvation methods use this kind of approximation for nonelectrostatic solvation with more refined atom-dependent surface tension terms^21,25,35,36^ or only for van der Waals contributions.^19,52,53^ The simpler approach using only one parameter of surface tension has also been used more recently.^54,55^ In the present study, our objective is to model stronger electrostatic interactions, and this approach is enough for this proposal. For more refined calculation in the future, cavity formation and attractive dispersion force contributions could be included separately.^56^

For the CCSA, the following equation is proposed in this work based on previous developments^10,49^
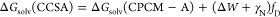
2where the first term on the right side is the continuum contribution for the solvation of the solute and the second term has two contributions inside the parentheses. The first term (Δ*W*) is the potential of mean force or “energy” for the process: A + S → AS, with A being the solute and S being the solvent molecule. This term is calculated with the CPCM method (only electrostatic) with a functional as X3LYP (without dispersion) and aims to evaluate the effect of the electrostatic interaction with an explicit solvent molecule. The γ_N_ term is a positive contribution, and in this work, it was evaluated empirically as γ_N_ = 1.0 kcal mol^–1^ to fit the solvation free energy. Both Δ*W* and γ_N_ terms result from the integral^10,49^
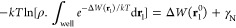
3

**Figure 1:**
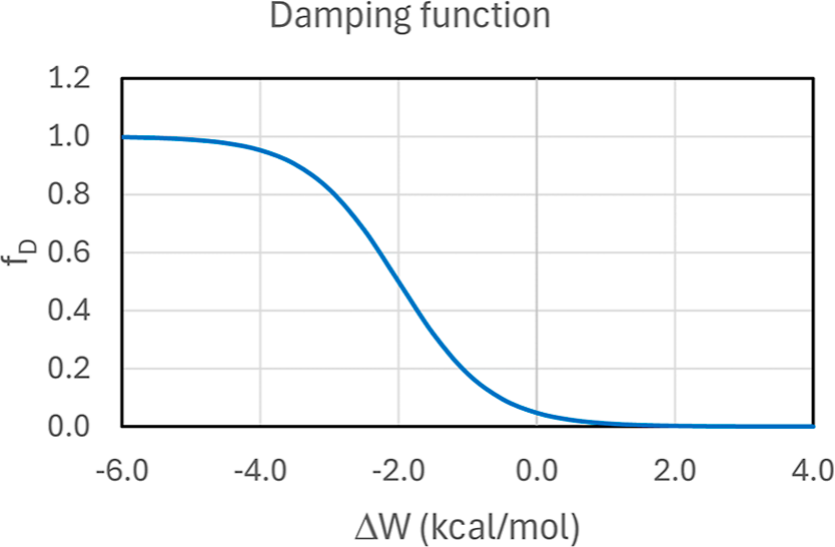
Form of the first multiplicative term of the damping function used in this work considering the second term as 1.

This integral corresponds to the integration in a potential well region where the solvent molecule is close to the solute and ρ corresponds to the density number of the solvent. The Δ*W*(*r*_1_^0^) is evaluated in the configuration of the AS complex leading to its most negative value. The multiplication factor f_D_ is a double damping function and is given by
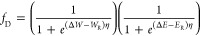
4where the constants were set to *W*_R_ = −2 kcal/mol, η = 3/2, and *E*_R_ = −12.0 kcal/mol. The aim of this damping function is to make the hybrid cluster-continuum approach general use for cations, anions, neutrals, and ion pairs. Because the CSM should work more accurately for neutrals and anions in acetonitrile, the first damping term transforms the Δ*G*_solv_ calculated by the CCSA to the Δ*G*_solv_ calculated by the pure continuum method when the Δ*W* became small, close to −2 kcal mol^–1^. The second multiplicative damping term allows the inclusion of explicit solvent correction only when the gas-phase interaction energy of the solute with the solvent (Δ*E*) is more negative than the reference value of *E*_R_ = −12.0 kcal/mol. Thus, the method has general use, and the effect of explicit solvent becomes important only when the explicit solute–solvent interaction is meaningful, reducing to the pure continuum solvation in the cases of weaker interactions. The behavior of the damping function is presented in Figure 1.

Aimed to show that the new CCSA method has superior performance compared to the CCQC approach, this method was also investigated for comparison. In the CCQC method, the solvation free energy of a solute A with one explicit solvent molecule S is given by^12^
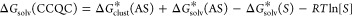
5

where the first term on the right side is the free energy for the formation of the AS complex in the gas phase (1 mol L^–1^ standard state), the second term is the solvation free energy of the AS complex, the third term is the solvation free energy solvent molecule, and the last term is related to the concentration of solvent molecules in solution, which is 1.75 kcal mol^–1^ for acetonitrile.^45^ It is worth observing that the solvation free energy of the solute A is calculated by eq 5 only if its value is more negative than the Δ*G*_solv_ by the pure continuum method. Equation 5 can be rewritten as
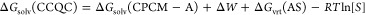
6where Δ*G*_vrt_(AS) is the vibrational, rotational, and translational contribution to the free energy for formation of the AS complex. Comparing eqs 4 and 6, we can obtain the relationship between these two hybrid methods, excluding the damping functions
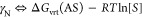
7

By this relation, the introduction of a fitting γ_N_ term allows the high entropy cost to bring the solute and solvent molecules together to be reduced because only when the solvent molecule is bound to the solute is relation eq 6 accurate. In future developments of the hybrid models, the integral in eq 3 can be calculated, and this γ_N_ term would be better evaluated with different values for each solute.

### 2.2 Theoretical Calculations

Geometry optimization was done in the gas phase using the X3LYP functional^57^ and the def2-SVP basis set^58^ (ma-def2-SVP for F, O, N, Cl, S, Br, I).^59^ The Stuttgart-Dresden ECP for the inner electrons was used for the iodine atom.^60^ The resolution of identity with the RIJCOSX method was used to accelerate the calculations. Harmonic frequency calculations were performed at this same level of theory. The solvation by the continuum was done by the CPCM model, as implemented in ORCA 5.0.3.^61,62^ In the case of the SMD model,^21^ the cavity used was the van der Waals surface, with the Gaussian charge scheme for representing the surface charges. This kind of surface produces close values of Δ*G*_solv_ for molecules in relation to the use of the SES cavity.^63^ In the case of CPCM-A computations, because solute–acetonitrile complexes were involved in some structures, the SES surface was used with the Gaussian charge scheme, using the atomic radii internally stored in the ORCA (H(1.10), C (1.70), N (1.55), O(1.52), Cl (1.75), S(1.80), Br(1.85), and I(1.98)) except for fluorine, which was fitted to 1.28 Å. A scale factor of 1.35 was applied for all the atoms; thus, the final atomic radii are multiplied by this factor. The choice of this value is based on studies pointing out that the scale factor close to 1.35 is adequate for anions in polar aprotic solvents.^64−66^ No additional fitting was performed for this parameter. In the determination of the α_A_ parameter, the solvation free energy of the neutral species was used to produce an optimal fitting, leading to α_A_ = −0.00531 kcal/Bohr^2^.

For some solutes, the gas-phase acidity (Δ*G*° for the process HA → A^–^ + H^+^, 1 mol L^–1^ standard state) was determined in this work by using a high level of theory, and the solvation free energy of the neutral HA species was determined by the SMD model. In these cases, the electronic energy for the acidity was calculated at the DLPNO–CCSD(T) level of theory^67^ (tight PNO) with the extended def2-TZVPP basis set (ma-def2-TZVPP for F, O, N, S). The X3LYP method with the same basis set used for geometry optimization was also used for the SMD calculations. In the case of some reactions used in the test of the solvation models, the accurate ωB97M-V functional^68^ with the same ma-def2-TZVPP basis set was used.

### 2.3 Solvation Data Set

The solvation data of ions used in this work is based on the bulk free energy scale reported by Carvalho and Pliego,^45^ which corresponds to Δ*G*_solv_* (*H*^+^) = −253.2 kcal mol^–1^ using a 1 mol L^–1^ standard state. This scale excludes the surface potential. The solvation free energies for F^–^, Cl^–^, Br^–^, and I^–^ anions were taken from that work. For a set of ions (A^–^ anions and BH^+^ cations), the most recent compilation of solvation data was used from Leonhard and co-workers,^50^ the DISSOLVE database. The data from DISSOLVE correspond to the anions A5 to A10 and A12 to A14 and the cations BH2 to BH22. In the case of A11, the gas-phase acidity reported in DISSOLVE was in error, with a value of 340.8 kcal mol^–1^, while the NIST database reports a value of 332.5 kcal mol^–1^.^69^ The calculation in this work determined a value of 333.1 kcal mol^–1^, which was used. The anions A15 to A22 correspond to more challenging polyfunctionalized anions, which were added to the data set. In these cases, the gas-phase acidity and solvation free energy of HA were calculated in this work, as described in the Theoretical Calculation section, and the p*K*_a_ values were taken from a recent compilation.^70^ The solvation free energy of the A^–^ anions was determined by the equation^45^
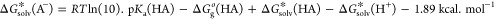
8

For the cations BH2 to BH22, the solvation free energy of BH^+^ cations was determined by the equation
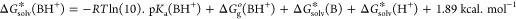
9taking the data from DISSOLVE and the proton solvation value used in this work. The 1.89 kcal mol^–1^ term is a correction from the 1 atm to 1 mol L^–1^ standard state because the gas-phase free energy for dissociation of the HA and BH^+^ species (Δ*G*_g_^*o*^ (HA) and Δ*G*_g_^o^ (BH^+^)) corresponds to the processes using the 1 atm standard state. For the protonate acetonitrile species BH1, the following equation was used^71^
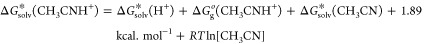
10with Δ*G*_g_^*o*^ (CH_3_ CNH^+^) = 179 kcal mol^–1^ taken from NIST, Δ*G*_solv_* (CH_3_ CN) = −4.19 kcal mol^–1^ based on the vapor pressure of pure acetonitrile taken from NIST, and *RT*ln[CH_3_ CN] = 1.75 kcal mol^–1^ based on the density of acetonitrile. These data were used to determine the experimental value of Δ*G*_solv_* (CH_3_ CNH^+^) = −74.8 kcal mol^–1^, included in the data set.

The solvation free energy values of 19 neutral species (mol1 to mol19) used to determine the atomic surface tension α_A_ were reported by Zanith and Pliego,^72^ corresponding to experimental values obtained from infinity dilution activity coefficient and vapor pressure. All of the solvation data used in this work are presented in Tables S1, S2, and S3 of the Supporting Information.

## 3 Results and Discussion

### 3.1 Solvation of Neutrals

**Figure 2:**
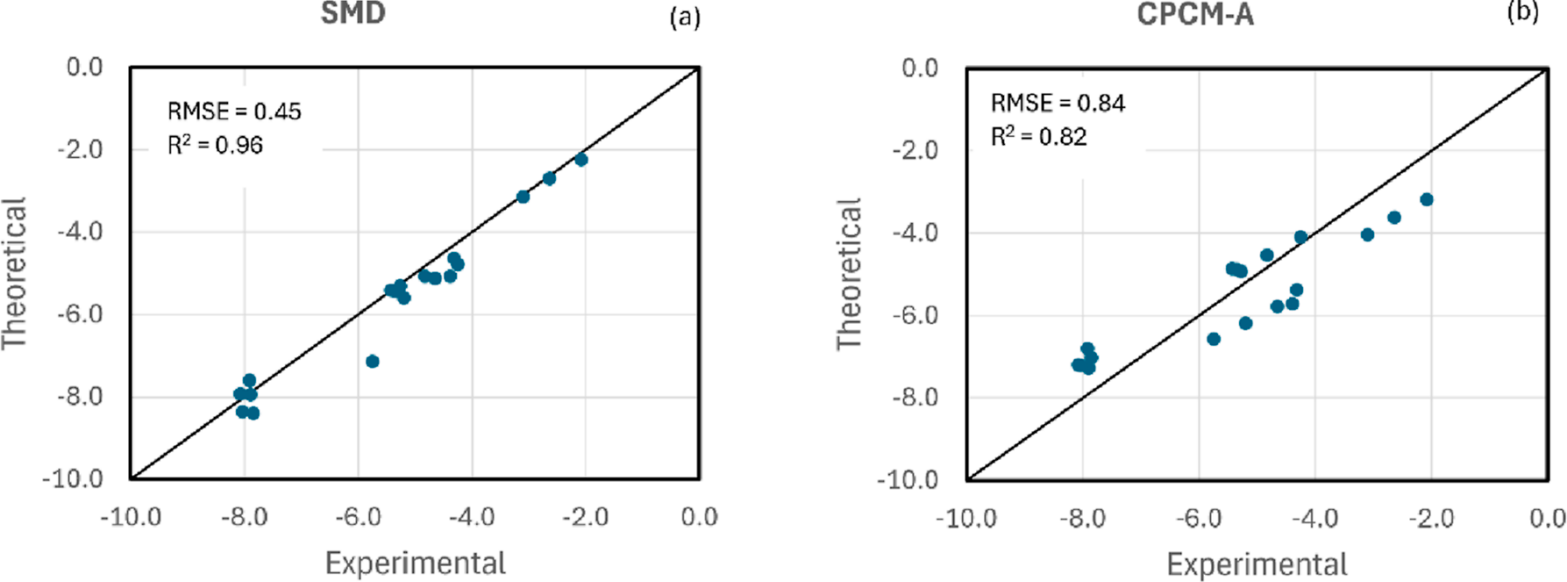
Test of the SMD and CPCM-A models for the solvation free energy of a set of 19 neutral species in acetonitrile. Units in kcal mol^–1^.

The first part of the analysis is a test of the SMD and CPCM-A models for neutral species. The results are presented in Figure 2 and in Table S1 of the Supporting Information. We can see that the SMD model performs very well, with an RMSE value of 0.45 kcal mol^–1^ and R^2^ = 0.96, even better than the previous report using force-field geometries with RMSE = 0.53 kcal mol^–1^.^72^ The PCM-A with a simple parameter of atomic surface tension is determined as α_A_ = −0.00531 kcal.mol^–1^. Bohr^–2^, performing worse as expected, with RMSE = 0.84 kcal mol^–1^. However, this is a reasonable performance considering that just one parameter for apolar solvation was fitted, and it is enough for testing the CCSA method for the challenging problem of ion solvation.

### 3.2 Solvation of Anions

**Figure 3:**
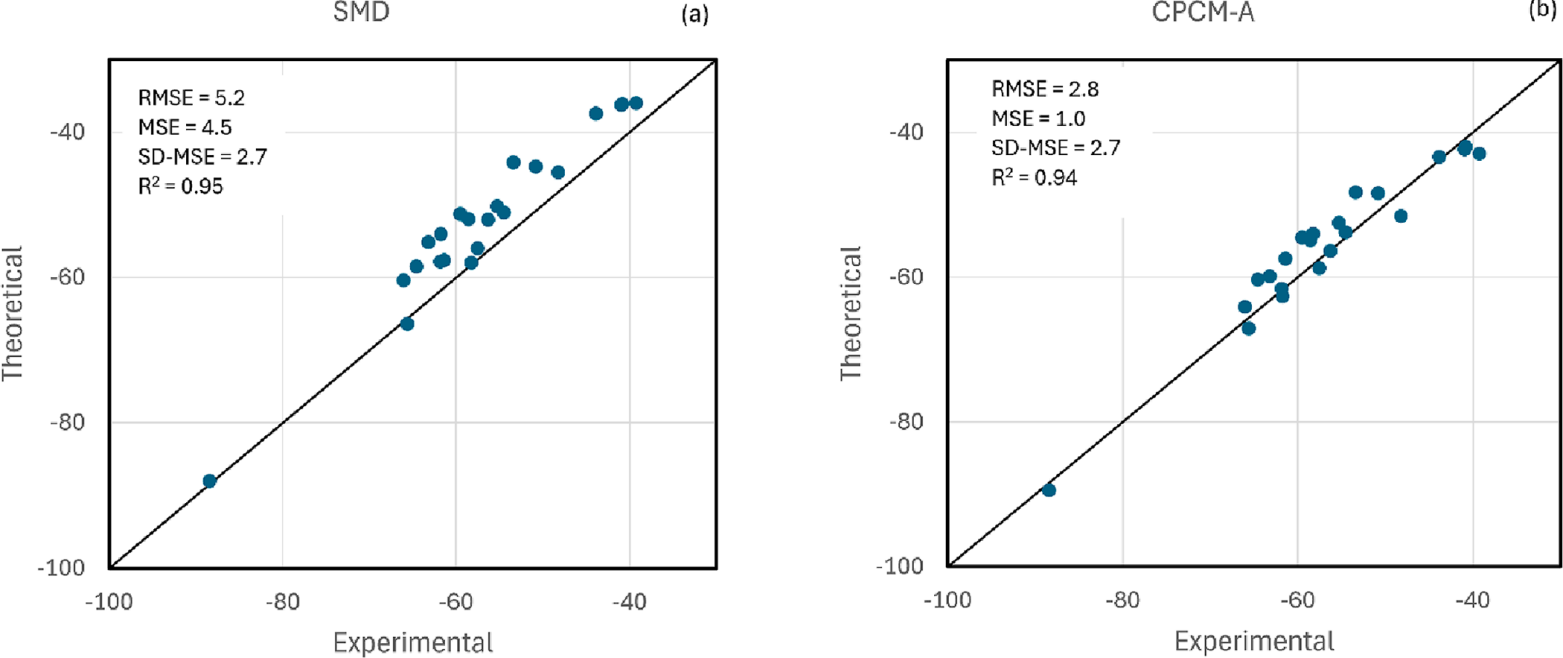
Test of the SMD and CPCM-A models for the solvation free energy of a set of 22 anions in acetonitrile including polyfunctionalized anions. Units in kcal mol^–1^.

We have long known that the solvation free energy of single anions can be calculated with reasonable accuracy in polar aprotic solvents using the PCM method with an adequate scaling factor of the atomic cavities.^65^ Thus, this work adds more complex, polyfunctionalized anions to the data set to provide a more challenging test of the solvation models. The results are given in Table S4 and Figure 3. The SMD model has a significant RMSE of 5.2 kcal mol^–1^. However, it has a very good value of R^2^ = 0.95. In part, this deviation is due to the fact that SMD has been parameterized considering that Δ*G*_solv_* (H^+^) = −260.2, or 7 kcal mol^–1^ more negative than the present value, indicating that the Δ*G*_solv_ of anions in this work should be shifted by 7 kcal mol^–1^ to make a fair comparison. To correct this difference, we have calculated the MSE and the SD-MSE, which correspond to 4.5 kcal mol^–1^ and 2.7 kcal mol^–1^, respectively. Thus, the SMD method performs well because of the reasonable value of SD-MSE. This parameter is a good indicator to measure the performance of the model for anion–molecule reactions because such a process involves differences in the solvation of anions.

When the simpler parameterized CPCM-A method is tested, which also involves the solvation of complex anions, the performance is impressive. Despite having many fewer parameters than SMD, the CPCM-A method performs as well as SMD, with RMSE = 2.8 kcal mol^–1^ and R^2^ = 0.94. The MSE is only 1.0 kcal mol^–1^ and the SD-MSE is 2.7 kcal mol^–1^. The highest deviation occurs for the polyfunctionalized anion A10, the 2,4-dinitro-phenoxide anion, with an error of 5.1 kcal mol^–1^ and a deviation of 4.1 kcal mol^–1^ from the MSE. For comparison, this anion also leads to the highest error of the SMD, 9.2 kcal mol^–1^, and a deviation from the MSE of 4.7 kcal mol^–1^. Thus, these results indicate that the CPCM-A model could be used for reliable modeling of anion–molecule reactions in the solution phase and should have a performance close to the highly parameterized SMD model.

### 3.3 Solvation of Cations

**Figure 4:**
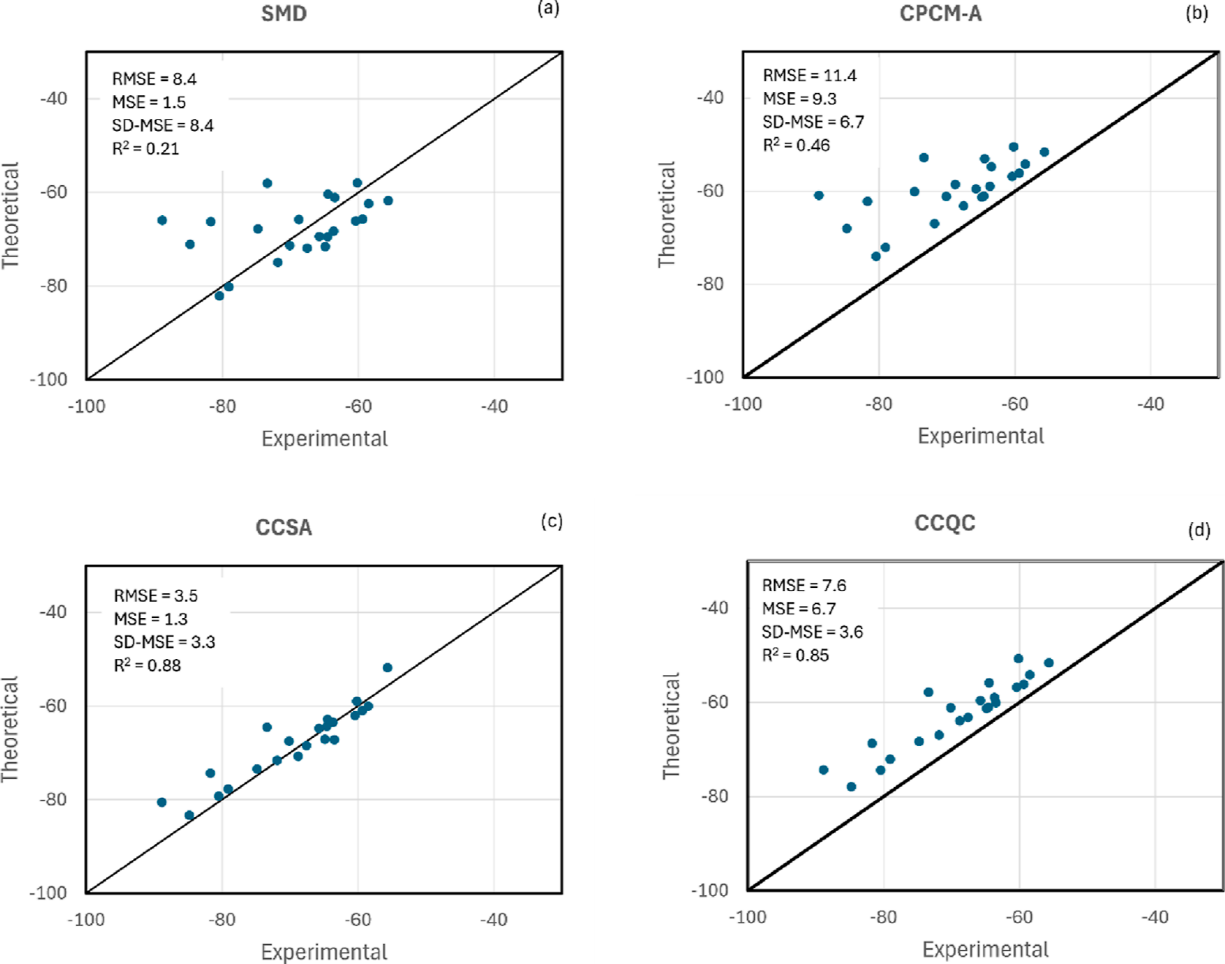
Test of the SMD, CPCM-A, CCSA, and CCQC models for the solvation free energy of a set of 22 cations in acetonitrile, including species with very strong solute–solvent interactions. Units in kcal mol^–1^.

Considering that cations should have stronger specific solute–solvent interactions than anions in acetonitrile, modeling cation solvation should be more challenging in this solvent. Further, it could be difficult to determine a single radius for each element able to work fine for cations and anions in the framework of continuum solvation. This idea has been verified in this work. Thus, the performance of the highly parameterized SMD model for the set of cations in acetonitrile investigated in this work is surprisingly poor as we can see in Figure 4. The MSE is only 1.5 kcal mol^–1^, but the corresponding SD-MSE has a very high value of 8.4 kcal mol^–1^, indicating a large dispersion of the error. The same high value is found for the RMSE, which is 8.4 kcal mol^–1^. This unexpectedly low performance is even more evident with a calculated R^2^ of 0.21, indicating a very low correlation between theoretical and experimental values.

In the case of the CPCM-A method, the poor performance is also verified, with an RMSE of 11.4 kcal mol^–1^ and a MSE of 9.3 kcal mol^–1^. Thus, the method is not able to provide accurate absolute solvation free energy of cations. However, considering the relative solvation free energy, the CPCM-A method surprisingly performs better than SMD, with an SD-MSE of 6.7 kcal mol^–1^. Analyzing the correlation between theoretical and experimental data, the value of R^2^ is 0.46, substantially superior to that of SMD. These findings are unexpected because a highly parameterized model such as SMD should present much better performance.

The next step of the analysis is to include an explicit solvent. The results for the CCSA method for cations are much superior to that for the pure continuum models, as we can see in Figure 4c. The RMSE is only 3.5 kcal mol^–1^, and the MSE becomes 1.3 kcal mol^–1^. Thus, the inclusion of an explicit solvent reduces the MSE from 9.3 kcal mol^–1^ in CPCM-A to only 1.3 kcal mol^–1^ in CCSA. The SD-MSE becomes 3.3 kcal mol^–1^, close to the uncertainty found for anion solvation with CPCM-A. The correlation also improves substantially, with R^2^ calculated to be 0.88, a good value. These results are very impressive because a simple inclusion of one acetonitrile molecule by the present model makes a huge difference in the performance. Thus, challenge processes such as AB → A^+^ + B^–^ can be studied by the present method, whereas it would lead to a very high error using SMD or CPCM-A. In the same way, cation–molecule reactions should also be much better described by the present CCSA approach, whereas a very poor performance is expected for SMD or CPCM-A. We can also notice that BH18, BH19, and BH20 are the most challenging solutes in acetonitrile solvent, leading to the highest deviations.

Another test of the present CCSA model is against the hybrid CCQC method, as presented in Figure 4d. We can notice that the inclusion of an explicit solvent in this approach also leads to a better performance in the calculation of Δ*G*_solv_ than that of the CPCM-A model. A point to observe is a more uniform description of the solvation of ions, with lower relative deviation. Nevertheless, the CCSA method outperforms the CCQC for all the parameters. Thus, whereas the CCSA method predicts Δ*G*_solv_ close to the experiments, with a MSE of 1.3 kcal mol^–1^, this parameter is 6.7 kcal mol^–1^ in the CCQC, indicating that this method is less accurate for the description of the formation of charged species in the solution phase. Even the SD-MSE and R^2^ parameters are improved in the CCSA method, with a value of R^2^ = 0.88 for CCSA and R^2^ = 0.85 for CCQC. These results indicate that the use of the constant γ_N_ parameter leads to more accurate relative Δ*G*_solv_ than including the effect of the frequency calculations.

**Figure 5:**
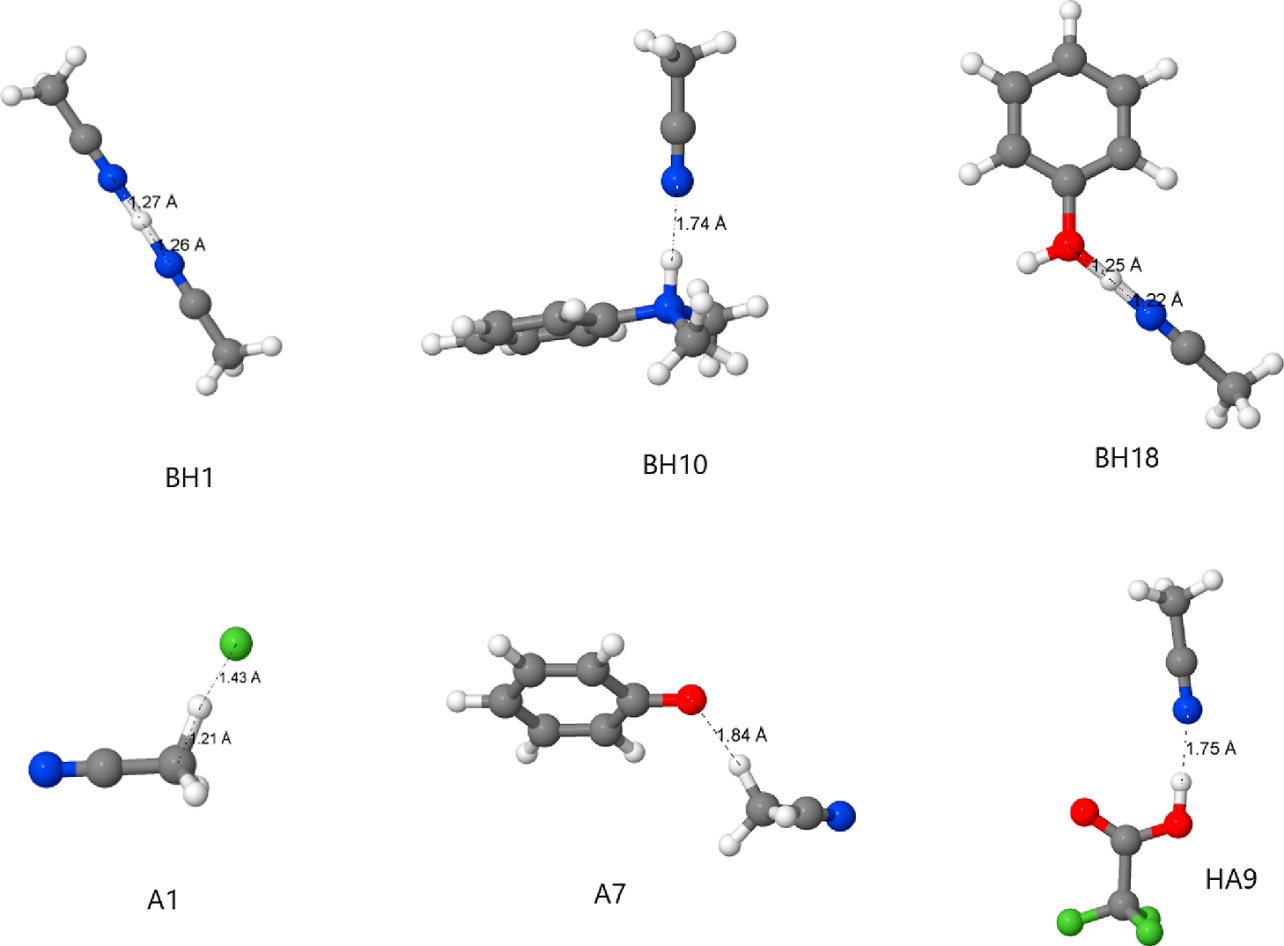
Some key optimized structures including explicit solvation by the acetonitrile molecule.

Aimed at better understanding how strong the solute–solvent interactions are and their effect on the solvation, the contributions to CCSA are presented in Table 1, and some key optimized structures are shown in Figure 5. The value of the Δ*W* for cations is as small as −1.6 kcal mol^–1^ for BH10 (protonated *N*,*N*-dimethylaniline) or as large as −20.7 kcal mol^–1^ for BH18 (protonated phenol). Whereas in BH10, the nitrogen of acetonitrile has a large distance from the hydrogen of the protonated solute (1.74 Å), in the case of BH18, the proton is essentially in the middle point between the nitrogen of acetonitrile and the oxygen of the phenol, with distances of 1.22 and 1.25 Å, respectively. Such a situation is impossible to capture by the continuum solvation and demands the inclusion of an explicit solvent. The error in the Δ*G*_solv_ in this case is 23.0 kcal mol^–1^ for SMD and 28.0 kcal mol^–1^ for CPCM-A and reduces considerably to 8.4 kcal mol^–1^ for CCSA. In the case of BH10, the error of SMD is −6.0 kcal mol^–1^ and becomes 3.9 kcal mol^–1^ for the CCSA method. Another interesting solute is BH1, a protonated acetonitrile. In this case, the SMD model has an error of 7.0 kcal mol^–1^, while in the CCSA method, the error is only 1.4 kcal mol^–1^. Thus, whereas the experimental difference of the Δ*G*_solv_ between BH10 and BH1 is 19.1 kcal mol^–1^, for the CCSA and SMD models, this value is 21.6 and 6.1 kcal mol^–1^, respectively. Observing Δ*E*, all of these species have interaction energy with one solvent molecule more negative than 15 kcal mol^–1^, indicating that the second damping function has a value of 1.0. Taking these results together, only the CCSA method can predict reliable proton transfer free energy between cationic protonated solutes and acetonitrile.

**Table 1:** Solvation Free Energy and the Effect of an Explicit Solvent^a^

	cations				
label	CPCM-A^b^	Δ*W*^c^	Δ*E*^d^	*f*_d_^e^	CCSA^f^
BH1	–60.09	–14.35	–34.53	1.00	–73.44
BH2	–74.00	–6.24	–29.84	1.00	–79.24
BH3	–66.95	–5.64	–25.86	1.00	–71.57
BH4	–63.15	–6.31	–24.97	1.00	–68.45
BH5	–58.95	–5.55	–22.07	1.00	–63.48
BH6	–59.55	–6.24	–22.80	1.00	–64.78
BH7	–61.06	–4.42	–22.54	0.97	–64.39
BH8	–56.13	–5.82	–21.24	1.00	–60.94
BH9	–61.11	–7.40	–24.84	1.00	–67.51
BH10	–51.59	–1.63	–15.24	0.36	–51.82
BH11	–56.82	–6.24	–22.54	1.00	–62.05
BH12	–54.15	–6.88	–22.70	1.00	–60.03
BH13	–72.05	–6.62	–28.36	1.00	–77.67
BH14	–67.98	–16.33	–40.12	1.00	–83.31
BH15	–54.73	–13.42	–29.13	1.00	–67.14
BH16	–58.57	–13.13	–31.10	1.00	–70.71
BH17	–50.43	–9.56	–23.41	1.00	–59.00
BH18	–60.87	–20.66	–38.20	1.00	–80.53
BH19	–62.19	–13.14	–32.22	1.00	–74.33
BH20	–52.82	–12.72	–26.96	1.00	–64.54
BH21	–53.04	–10.80	–18.82	1.00	–62.83
BH22	–61.30	–6.76	–24.02	1.00	–67.05

	anions				
label	CPCM-A^b^	Δ*W*^c^	Δ*E*^d^	*f*_d_^e^	CCSA^f^
A1	–89.48	2.75	–26.07	0.00	–89.48
A2	–67.13	0.00	–14.04	0.05	–67.08
A5	–64.13	–0.76	–16.77	0.14	–64.10
A7	–57.47	–2.17	–14.53	0.55	–58.12
A15	–54.05	–0.86	–12.97	0.12	–54.03
A17	–43.43	–1.03	–8.87	0.00	–43.43
A18	–51.60	–1.08	–12.21	0.12	–51.61

	neutrals				
label	CPCM-A^b^	ΔW^c^	Δ*E*^d^	*f*_d_^e^	CCSA^f^
B14	–5.11	–3.95	–5.47	0.00	–5.11
B16	–6.63	–0.72	–3.63	0.00	–6.63
HA9	–7.15	–8.25	–11.14	0.22	–8.73
HA13	–10.84	–7.55	–9.93	0.04	–11.12

a – Units in kcal mol^–1^, 298 K.

b – Solvation free energy by the CPCM-A method.

c – Potential of mean force contribution.

d – Solute–solvent interaction energy.

e – Damping term.

f – Final CCSA.

Observing the effect of Δ*W* on anions, we can note that the effect is small, with the most negative value of the sample in Table 1 corresponding to the phenoxide ion (A7). Even for the fluoride ion (A1), although there is a meaningful interaction considering the F–H distance in Figure 5, the value of the Δ*W* is small and positive, resulting in no alteration of the Δ*G*_solv_. Considering Δ*E*, the values are less negative than those observed for cations, as expected. However, some values are as small as −8.87 kcal mol^–1^ as observed for A17, a species with a high charge dispersion. The damping function makes the explicit solvent correction minimal, and both the CPCM-A and CCSA methods have essentially the same values of Δ*G*_solv_.

In the case of neutrals, four species were analyzed: methanol (B14), acetone (B16), trifluoroacetic acid (HA9), and methanesulfonic acid (HA13). For acetone, Δ*W* is small and negative but has a higher correction for methanol and is even more negative for trifluoroacetic acid, amounting to −8.3 kcal mol^–1^. This finding can be explained by the strong hydrogen bond between fluorinated carboxylic acid and the acetonitrile molecule. The strong methanesulfonic acid has also a meaningful negative value of Δ*W*, −7.55 kcal mol^–1^. When we look at the Δ*E* parameter, it is possible to notice that the corresponding values are some kcal mol^–1^ more negative than the Δ*W*. Only the strong acids HA9 and H13 have enough negative values of Δ*E* to make some small corrections to the continuum solvation.

### 3.4 Test of the Model for Some Reactions

In this section, a test of the CCSA model and a comparison with the SMD method were done for some reactions leading to the formation of charged species in solution. Three reactions of proton transfer are presented in Table 2, compared with the experimental data obtained from p*K*_a_ values. It is worth observing that in the application of the CCSA method, only one explicit acetonitrile can be included in each side of the chemical equation to make the approach consistent. Looking at the results, we can observe that the reactions involve a huge variation of energy calculated by the ωB97M-V functional, indicating that an accurate solvation model is needed to predict accurate solution phase free energies. The first reaction is the ionization of benzoic acid and formation of protonated piperidine with an experimental value of Δ*G*_sol_ = 1.8 kcal mol^–1^. In this case, both the SMD and CCSA perform very well, with the CPCM-A method presenting a larger deviation. In the second case, the benzoic acid protonates methanol with experimental Δ*G*_sol_ = 25.0 kcal mol^–1^. In this difficult case, only the CCSA method predicts a reasonable value of Δ*G*_sol_ = 29.1 kcal mol^–1^, with a deviation of 4.1 kcal mol^–1^. The SMD method performs poorly with a deviation of 17.2 kcal mol^–1^. The third reaction is the protonation of the carbonyl of the benzoic acid by another benzoic acid. This self-ionization reaction has an experimental value of Δ*G*_sol_ = 28.2 kcal mol^–1^. Again, only the CCSA method predicts a reasonable value of Δ*G*_sol_, with a deviation of 4.2 kcal mol^–1^ from the experimental data. The SMD model presents a deviation of 13.0 kcal mol^–1^. Considering these results, we can claim that the new method is much more reliable for modeling these difficult reactions in the solution phase when charged species are formed or consumed.

**Table 2:** Test of the Solvation Models for Some Reactions in Acetonitrile Solution^a^

	ΔΔ*G*_solv_^b^						Δ*G*_sol_^c^		
reaction	ωB97M-V^d^	Δ*G*_vrt_^e^	CPCM-A	SMD	CCSA	CPCM-A	SMD	CCSA	exp^f^
PhCOOH + piperidine→PhCOO^–^ + piperidineH^+^	111.95	0.83	–104.54	–111.07	–109.77	8.2	1.7	3.0	1.8
PhCOOH + MeOH→PhCOO^–^ + MeOH_2_^+^	160.57	–0.86	–115.26	–117.53	–130.59	44.4	42.2	29.1	25.0
PhCOOH + PhCOOH→PhCOO^–^ + PhCOOH_2_^+^	142.31	–1.04	–97.18	–100.05	–108.89	44.1	41.2	32.4	28.2

a – Units in kcal mol^–1^, 298 K.

b – Solvation free energy variation by the three methods described in the text.

c – Solution phase free energy of reaction.

d – Electronic energy calculated with the ma-def2-TZVPP basis set.

e – Vibrational, rotational, and translational contributions to the free energy.

f – Experimental values obtained from p*K*_a_ data: Δ*G*_sol_ = 1.364.(p*K*_a_(HA) – p*K*_a_(BH^+^)).

## 4 Conclusions

This work presents further development and testing of a new hybrid cluster-continuum solvation approach for modeling ion solvation. The results show that in acetonitrile solvent, whereas the inclusion of an explicit solvent is not needed for the solvation of anions, in the case of cations, explicit solvation by one acetonitrile molecule is critical for obtaining reliable values of solvation free energies. The new hybrid model considerably outperforms the highly parameterized SMD model for the solvation of cations and presents a similar performance for anions. The CCSA method also has better performance than the hybrid CCQC approach, with the advantage that no frequency calculation is needed. This new model can be utilized for predicting the free energy of ionic reactions in the solution phase. Further refinement of the nonelectrostatic solvation and automation of the method by placing an explicit solvent in the most important site of the solute could make this approach a useful, practical, and relatively accurate solvation method.

## Supporting Information Available

Solvation free energy of neutral molecules, anions, and cations in acetonitrile solvent and performance of the models for anions and cations in acetonitrile solvent (PDF)
Coordinates of the optimized structures (ZIP)
