# Supplementary material for: Hybrid Cluster-Continuum Method for Single-Ion Solvation Free Energy in Acetonitrile Solvent
Source: J Phys Chem A. 2024 Jul 25;128(31):6440–9. doi: 10.1021/acs.jpca.4c03593 (PMC11317976; doi:10.1021/acs.jpca.4c03593)
Supplement: Supplementary file 1 — jp4c03593_si_001.pdf [file jp4c03593_si_001.pdf]

Supporting Information

Hybrid Cluster-Continuum Method for Single-Ion Solvation Free Energy in Acetonitrile Solvent

Josefredo R. Pliego Jr.*

Departamento de Ciências Naturais, Universidade Federal de São João del-Rei
36301-160, São João del-Rei, MG, Brazil.

Table S1: Solvation free energy of neutral molecules in acetonitrile solvent.[a]

		ΔG_{solv}		
**Label**	**Molecule**	**Exp.**[b]	**SMD**[c]	**CPCM-A**[d]
mol1	ethanol	-4.32	-4.63	-5.37
mol2	1-propanol	-4.65	-5.12	-5.78
mol3	2-propanol	-4.39	-5.06	-5.71
mol4	n-butanol	-5.20	-5.60	-6.19
mol5	benzene	-4.25	-4.78	-4.09
mol6	toluene	-4.83	-5.07	-4.53
mol7	ethylbenzene	-5.36	-5.44	-4.87
mol8	o-xylene	-5.43	-5.41	-4.87
mol9	m-xylene	-5.28	-5.33	-4.92
mol10	p-xylene	-5.27	-5.31	-4.93
mol11	n-pentane	-2.08	-2.24	-3.18
mol12	n-hexane	-2.64	-2.69	-3.61
mol13	n-heptane	-3.10	-3.14	-4.03
mol14	o-cresol	-7.92	-7.60	-6.80
mol15	m-cresol	-7.90	-7.94	-7.27
mol16	p-cresol	-8.08	-7.93	-7.20
mol17	o-toluidine	-7.85	-8.40	-7.03
mol18	m-toluidine	-8.04	-8.37	-7.22
mol19	γ-picoline	-5.75	-7.14	-6.57
**RMSE**			0.45	0.84

a – Units in kcal mol^{-1}, 298 K. b – Experimental data from reference [1]. c – Solvation free energy calculated with the SMD model and X3LYP functional with the def2-SVP basis set (ma-def2-SVP for O and N). Same method used for geometry optimization. d – Solvation free energy calculated by the CPCM-A method with parameters defined in the main text and also using X3LYP functional.

Table S2: Solvation free energy of anions in acetonitrile solvent.[a]

Label	Anion	$\Delta G_g^o(HA)^b$	$\Delta G^*_{solv}(HA)^b$	$pK_a(HA)^c$	$\Delta \mathbf{G_{solv}}^*(\mathbf{A^-})^d$
A1	F^-				-88.4[e]
A2	Cl^-				-65.6[e]
A3	Br^-				-61.7[e]
A4	I^-				-57.5[e]
A5	CH_3COO^-	342.4	-7.0	23.5	-66.0
A6	$PhCOO^-$	334	-10.1	20.7	-64.6
A7	PhO^-	343.4	-9.1	29.2	-61.4
A8	o-NO_2-Ar-O^-	332.6	-8.5	22.9	-58.6
A9	CF_3COO^-	317.5	-7.4	12.7	-56.3
A10	2,4-$(NO_2)_2$-Ar-O^-	315.9	-11.7	16.8	-53.4
A11	m-CF_3-Ar-O^-	333.1[f]	-9.6	26.5	-55.2
A12	p-NO_2-Ar-COO^-	323	-13.3	18.7	-59.5
A13	$CH_3SO_3^-$	313	-13.7	9.97	-61.8
A14	NO_3^-	319.4	-7.2	8.9	-63.2
A15	indolide$^-$	344.44[f]	-9.50	32.57	-58.2
A16	$(CF_3)_5$-Ar-NH^-	318.05[f]	-7.69	24.57	-40.9
A17	$(C_6F_5)_2N^-$	320.12[f]	-7.70	23.98	-43.8
A18	$(CF_3)_3CO^-$	325.54[f]	-1.89	20.5	-48.2
A19	$(CF_3)_5$-Ar-O^-	298.11[f]	-6.68	10.46	-39.2
A20	$(CF_3)_5$-Ar-S^-	299.58[f]	-6.33	10.12	-40.8
A21	$(CN)_3C^-$	297.29[f]	-11.61	5.00	-50.8
A22	2,4-$(NO_2)_2$-C_6H_3-SO_3^-	296.32[f]	-14.88	3.96	-54.5

a – Units in kcal mol^{-1}, 298 K. b – Theoretical data from reference [2]. c – Experimental data from reference [3]. d – Solvation free energy calculated using $\Delta G^*_{solv}(H^+)$ = -253.2 kcal mol^{-1}. e – From reference [4]. f – calculated in this work.

Table S3: Solvation free energy of cations in acetonitrile solvent.[a]

Label	Cation	$\Delta G_g^o(BH^+)$[b]	$\Delta G^*_{solv}(B)$[b]	$pK_a(BH^+)$[c]	**$\Delta G_{solv}^*(BH^+)$[d]**
BH1	CH_3CNH^+				-74.8[e]
BH2	NH_4^+	195.9	-2.6	16.46	-80.5
BH3	$CH_3NH_3^+$	207.4	-2.9	18.37	-71.9
BH4	$CH_3CH_2CH_2NH_3^+$	212.6	-3.7	18.44	-67.6
BH5	$(CH_3)_3CNH_3^+$	216.4	-4.1	18.1	-63.7
BH6	CyclohexanamineH$^+$	216	-5.4	18.36	-65.8
BH7	dimethylamineH$^+$	215.6	-2.9	19.03	-64.6
BH8	diethylamineH$^+$	221.1	-3.6	18.75	-59.4
BH9	anilineH$^+$	203.7	-8	10.64	-70.1
BH10	$Ph(NH^+)(CH_3)_2$	218.7	-7.4	11.47	-55.7
BH11	piperidineH$^+$	221.9	-4.6	19.35	-60.4
BH12	piridineH$^+$	215.8	-5.9	12.53	-58.5
BH13	hydrazineH$^+$	199.8	-4.9	16.61	-79.1
BH14	$CH_3OH_2^+$	173.8	-4	2.4	-84.8
BH15	$CH_3CH_2(OH^+)CH_2CH_3$	191.3	-3.2	0.2	-63.5
BH16	acetoneH$^+$	187.1	-4.7	-0.1	-68.8
BH17	1-phenylethanoneH$^+$	198.8	-7.8	-0.1	-60.2
BH18	$PhOH_2^+$	172.1	-9.1	0.4	-88.9
BH19	$CH_3C(OH)_2^+$	178.1	-7	1.1	-81.7
BH20	$PhC(OH)_2^+$	188.0	-10.1	0	-73.4
BH21	BenzamideH$^+$	203.8	-11.8	3.8	-64.5
BH22	morpholineH$^+$	215.2	-6.1	16.61	-64.9

a – Units in kcal mol^{-1}, 298 K. b – Theoretical data from reference [2]. c – Experimental data from reference [3]. d – Solvation free energy calculated using $\Delta G^*_{solv}(H^+)$ = -253.2 kcal mol^{-1}. e –Calculation discussed in the text.

Table S4: Performance of the models for anions and cations in acetonitrile solvent.[a]

Anions				Cations					
Label	Exp.	SMD	CPCM-A	Label	Exp.	SMD	CPCM-A	CCSA	CCQC
A1	-88.4	-88.1	-89.5	BH1	-74.8	-67.8	-60.1	-73.4	-68.3
A2	-65.6	-66.4	-67.1	BH2	-80.5	-82.1	-74.0	-79.2	-74.4
A3	-61.7	-54.1	-62.7	BH3	-71.9	-75.0	-66.9	-71.6	-66.9
A4	-57.5	-56.0	-58.8	BH4	-67.6	-71.9	-63.1	-68.5	-63.1
A5	-66.0	-60.4	-64.1	BH5	-63.7	-68.3	-59.0	-63.5	-59.0
A6	-64.6	-58.6	-60.4	BH6	-65.8	-69.4	-59.6	-64.8	-59.6
A7	-61.4	-57.7	-57.5	BH7	-64.6	-69.4	-61.1	-64.4	-61.1
A8	-58.6	-52.0	-54.9	BH8	-59.4	-65.7	-56.1	-60.9	-56.1
A9	-56.3	-52.1	-56.5	BH9	-70.1	-71.3	-61.1	-67.5	-61.1
A10	-53.4	-44.2	-48.3	BH10	-55.7	-61.7	-51.6	-51.8	-51.6
A11	-55.2	-50.2	-52.6	BH11	-60.4	-66.1	-56.8	-62.1	-56.8
A12	-59.5	-51.3	-54.6	BH12	-58.5	-62.4	-54.2	-60.0	-54.2
A13	-61.8	-57.9	-61.6	BH13	-79.1	-80.2	-72.1	-77.7	-72.1
A14	-63.2	-55.2	-59.9	BH14	-84.8	-71.0	-68.0	-83.3	-78.0
A15	-58.2	-58.0	-54.0	BH15	-63.5	-61.1	-54.7	-67.1	-60.1
A16	-40.9	-36.4	-42.4	BH16	-68.8	-65.7	-58.6	-70.7	-63.9
A17	-43.8	-37.5	-43.4	BH17	-60.2	-58.0	-50.4	-59.0	-50.7
A18	-48.2	-45.6	-51.6	BH18	-88.9	-65.9	-60.9	-80.5	-74.3
A19	-39.2	-36.1	-43.0	BH19	-81.7	-66.3	-62.2	-74.3	-68.7
A20	-40.8	-36.2	-42.0	BH20	-73.4	-58.0	-52.8	-64.5	-57.9
A21	-50.8	-44.8	-48.5	BH21	-64.5	-60.4	-53.0	-62.8	-55.8
A22	-54.5	-51.1	-53.9	BH22	-64.9	-71.5	-61.3	-67.1	-61.3
RMSE		5.2	2.8	RMSE		8.4	11.4	3.5	7.6
MSE		4.5	1.0	MSE		1.5	9.3	1.3	6.7
SD-MSE		2.7	2.7	SD-MSE		8.4	6.7	3.3	3.6

a – Units in kcal mol^{-1}, 298 K.

References

(1) Zanith, C. C.; Pliego, J. R., Jr., Performance of the smd and sm8 models for predicting solvation free energy of neutral solutes in methanol, dimethyl sulfoxide and acetonitrile. *J. Comput. Aided Mol. Des.* **2015,** *29*, 217-224.

(2) Nevolianis, T.; Baumann, M.; Viswanathan, N.; Kopp, W. A.; Leonhard, K., Dissolve: Database of ionic solutes’ solvation free energies. *Fluid Phase Equilibr.* **2023,** 113801.

(3) Kütt, A.; Tshepelevitsh, S.; Saame, J.; Lõkov, M.; Kaljurand, I.; Selberg, S.; Leito, I., Strengths of acids in acetonitrile. *European Journal of Organic Chemistry* **2021,** *2021*, 1407-1419.

(4) Carvalho, N. F.; Pliego, J. R., Cluster-continuum quasichemical theory calculation of the lithium ion solvation in water, acetonitrile and dimethyl sulfoxide: An absolute single-ion solvation free energy scale. *Phys. Chem. Chem. Phys.* **2015,** *17*, 26745-26755.
